# Comparative Study on Noise Reduction Effect of Fiber Optic Hydrophone Based on LMS and NLMS Algorithm

**DOI:** 10.3390/s20010301

**Published:** 2020-01-05

**Authors:** Zhihua Yu, Yunfei Cai, Daili Mo

**Affiliations:** 1 School of Automation, China University of Geosciences, Wuhan 430074, China; 2 Hubei Key Laboratory of Advanced Control and Intelligent Automation for Complex Systems, Wuhan 430074, China

**Keywords:** fiber optic hydrophone, 3 × 3 coupler, least mean square (LMS), normalized least mean square (NLMS), adaptive filtering

## Abstract

Adaptive filtering has the advantages of real-time processing, small computational complexity, and good adaptability and robustness. It has been widely used in communication, navigation, signal processing, optical fiber sensing, and other fields. In this paper, by adding an interferometer with the same parameters as the signal interferometer as the reference channel, the sensing signal of the interferometric fiber-optic hydrophone is denoised by two adaptive filtering schemes based on the least mean square (LMS) algorithm and the normalized least mean square (NLMS) algorithm respectively. The results show that the LMS algorithm is superior to the NLMS algorithm in reducing total harmonic distortion, improving the signal-to-noise ratio and filtering effect.

## 1. Introduction

In many studies of fiber optic hydrophone, the source of noise in the system is mainly the noise generated by the light source and other optical transmissions, in addition to environmental factors such as vibration, bending, and temperature changes. It causes random changes in parameters such as length and refractive index, and finally introduces strong background noise in the system [1,2,3]. These noises not only reduce the detection capability and sensitivity of fiber-optic hydrophones for weak signals, but also greatly limit the development of large-scale array and the long-distance transmission of fiber-optic hydrophones. In existing research, many scholars have achieved different noise reduction effects. In 2015, Romashk R. V. et al. [4] proposed and studied the implementation of an adaptive fiber-optic hydrophone with a membrane fiber sensor as the sensitive component. The experimental results show that the scheme improves the noise immunity and sensitivity of the hydrophone. In 2016, Vikash Chandra et al. [5] proposed and tested a two-channel Mach–Zehnder interferometer to eliminate the intensive disturbance noise, which can improve the quality of the demodulated signal by 3 dB. In 2018, Fei Liu et al. [6] reduced the noise floor of the heterodyne interferometric fiber sensor by adjusting the parameters of the acousto-optic modulator. The experimental results show that the phase noise caused by the acousto-optic modulator can be reduced to −130 dB at the optimal driving power. However, most of the above studies use the traditional signal detection method based on the phase-generated carrier (PGC) and the noise reduction method that changes the optical structure and instrument parameters. Not only does it have a limited signal detection range, but also poor adaptability and robustness that additionally causes other noise such as random noise and circuit noise.

Adaptive filtering technology mainly adjusts the filter parameters through the adaptive algorithm to achieve the best estimation of the system noise, and then achieves the optimal noise cancellation effect. In recent years, adaptive filtering technology has been widely used in the fields of wireless communication, voice signal processing, radar navigation, etc. due to its small amount of calculation and it being suitable for real-time processing, and has also begun to be applied to the field of optical fiber sensing. Therefore, in this paper, by adding a reference interferometer with the same parameters as the signal interferometer and adding an acoustic shielding tank to prevent external interference, the demodulation method using 3 × 3 coupler [7] is used to increase the detection range and reduce the system noise. Finally, the adaptive filtering technology is used to reduce the noise of the interferometric fiber optic hydrophone. From the perspective of real-time processing and convergence speed, the least mean square algorithm (LMS) and the normalized least mean square algorithm (NLMS) are used to compare the noise reduction effects of the two adaptive algorithms in the fiber optic hydrophone.

## 2. System Structure of Interferometric Fiber Optic Hydrophone

Commonly used signal detection schemes include the interferometric demodulation method using the 3 × 3 coupler and signal detection method based on the phase-generated carrier (PGC). However, when the two schemes are compared with each other, the 3 × 3 coupler demodulation scheme not only has a simple principle and a large signal detection range, but also reduces phase noise, random noise, and the requirements of the laser whilst not requiring carrier signal modulation [8].

**Figure 1:**
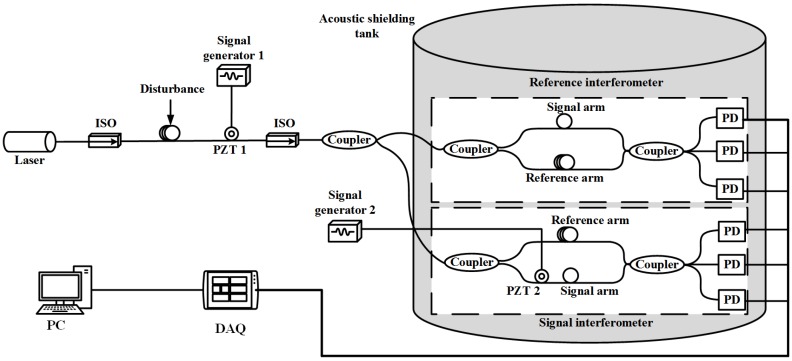
System structure of noise generation and signal detection of interferometric fiber optic hydrophone.

The system structure of noise generation and signal detection of interferometric fiber optic hydrophone is shown in Figure 1.

The output end of the laser is connected to the isolator to prevent the reflected light from returning in the subsequent optical path. Adding a disturbance to the optical fiber can change the polarization state of the optical signal to generate polarization noise. Winding the fiber on the piezoelectric ceramic (PZT 1), the noise signal generated by the signal generator 1 is applied to the PZT 1 to generate phase modulation noise. Then, the optical signal containing the noise signal is divided into two beams of light intensity with a ratio of 1/1 through a 3 dB 2 × 2 coupler into the signal interferometer and the reference interferometer, respectively. The two interferometers are Mach–Zehnder interferometers with unequal arms, with an arm difference of 10 m and placed in an acoustic shielding tank to isolate interference from external signals. Wherein, the signal arm of the signal interferometer is wound on the PZT 2 to add the valid signal, and the signal generator 2 is used to generate the valid signal. The two beams interfere in a 3 × 3 coupler and are divided into three paths. In the case of no loss of the coupler, the three beams of the 3 × 3 coupler output are equal in intensity and the phase difference between them is equal, both being 120${}^{\circ }$. The photodetector is used to detect the change of the interferometric intensity at the terminal. After the three optical signals are photoeletrically converted and can be expressed as:

(1) $$\left\{\begin{array}{c} I_{1}=A+B\cos\left[\varphi \left(t\right)-2\pi /3\right]\\ I_{2}=A+B\cos\varphi \left(t\right)\\ I_{3}=A+B\cos\left[\varphi \left(t\right)+2\pi /3\right] \end{array}\right.$$

Among them, *A* and *B* are the amplitudes of the offset and AC components of the interferometric signal, $\varphi \left(t\right)=\varphi_{s}+\varphi_{n}$, $\varphi_{s}$ is the valid signal, and $\varphi_{n}$ is the noise.

**Figure 2:**
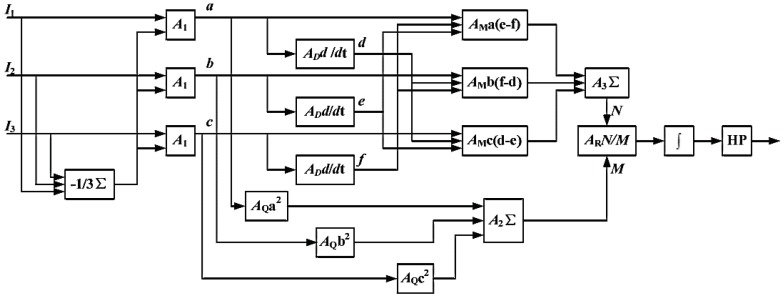
Schematic diagram of interferometric demodulation method using the 3 × 3 coupler.

The schematic diagram of interferometric demodulation method using the 3 × 3 coupler is shown as Figure 2. In the figure, $A_{1}$, $A_{2}$, and $A_{3}$ are adders, and $A_{D}$, $A_{M}$, $A_{Q}$, and $A_{R}$ are differentiators, multipliers, squarers, and dividers, respectively.

Using the phase difference of the three-channel signals, we get:

(2) $$N=\frac{3\sqrt{3}}{2}\cdot B^{2}\cdot \varphi^{\prime }\left(t\right).$$

The value *B* is unstable in practice, and is affected by changes in the intensity of the light source and changes in the polarization state of the interferometer fiber. The sum of squares of the three input signals after eliminating the offset component *A* can be used to eliminate *B*:

(3) $$M=a^{2}+b^{2}+c^{2}=\frac{3}{2}B^{2}.$$

Finally, after *N* and *M* are passed through the divider, the phase noise caused by the environmental disturbance is eliminated by the integral and high-pass filter to obtain the signal φ(t). Under the condition that the fiber parameters are basically constant, the phase difference between the signal arm and the reference arm is proportional to the measured signal [9]. The signal is demodulated by the 3 × 3 coupler demodulation algorithm to obtain the signal of the signal interferometer (including the valid signal and noise), and the signal of the reference interferometer (only noise).

## 3. Adaptive Filtering

In the working environment of a fiber optic hydrophone, a variety of noise signals are inevitably present and vary with time and space. This makes fixed filters designed according to prior knowledge not functioning properly or even failing. However, adaptive filtering can adjust its own parameters according to changes in the external environment to achieve the effect of noise reduction [10].

**Figure 3:**
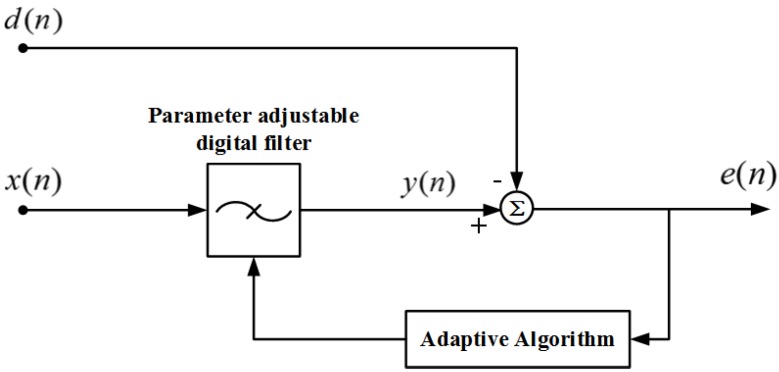
Adaptive filter schematic.

The schematic diagram of adaptive filtering is shown in Figure 3. The input signal x(n) generates an output signal y(n) via a parameter tunable digital filter, and the signal d(n) is compared with y(n) to obtain a difference signal e(n). The filter parameters are adjusted according to e(n) by an adaptive algorithm, and finally the mean square value of e(n) is minimized [11]. The scheme does not need to know the statistical characteristics of the input signal and noise in advance, and gradually estimates the required statistical characteristics in the process of signal processing, and automatically adjusts its own parameters based on the characteristics to achieve the optimal filtering effect. Once the statistical characteristics of the input signal change, it automatically tracks the changes and adjusts the parameters to optimize filter performance.

In this system, the input signals x(n) and d(n) respectively represent the output signals of the reference interferometer and the signal interferometer after demodulation by the 3 × 3 coupler. At a certain moment *k*, the output signal of the noise signal x(k) after the adaptive filtering is y(k), d(k)=s(k)+n(k) is the original signal, s(k) is the valid signal, n(k) is the noise, and the expected value of the square of the difference signal e(k) is obtained.

(4) $$E\left[e{\left(k\right)}^{2}\right]=E\left[s{\left(k\right)}^{2}\right]+E\left[{\left(n\left(k\right)-y\left(k\right)\right)}^{2}\right]+2E\left[s\left(k\right)\left(n\left(k\right)-y\left(k\right)\right)\right]$$

when $E[e{\left(k\right)}^{2}]$ is minimum, $E[{\left(n\left(k\right)-y\left(k\right)\right)}^{2}]$ also reaches a minimum, where y(k) is the best mean square estimate of noise n(k), and system output e(k) is the best mean square estimate of the valid signal s(k).

There are many kinds of adaptive algorithms, which are mainly used to adjust the adaptive filter parameters according to the input signal to obtain the desired signal. However, considering the real-time processing and convergence speed, the LMS algorithm and the NLMS algorithm are selected.

LMS algorithm: Take a single input as an example, let the filter length be *L*. At a certain moment *k*, the coefficient of the adaptive filter is $W_{k}={\left[\omega_{1},\omega_{2},\cdots ,\omega_{L}\right]}^{T}$, and the input of reference channel is $X_{k}={\left[n_{0}\left(1\right),n_{0}\left(2\right),\cdots ,n_{0}\left(L\right)\right]}^{T}$, then the output is:

(5) $$y\left(k\right)=W_{k}^{T}X_{k}$$

(6) $$e\left(k\right)=d\left(k\right)-W_{k}^{T}X_{k}.$$

To minimize the value of $E[e{\left(k\right)}^{2}]$, zero the gradient of $W_{k}$ to get the best value of $W_{k}$. In practical applications, gradient calculations use instantaneous estimates [12]:

(7) $$\nabla_{k}=\frac{\partial e{\left(k\right)}^{2}}{\partial W_{k}^{T}}=2e\left(k\right)\frac{\partial e(k)}{\partial W_{k}^{T}}=-2e\left(k\right)X_{k}$$

In order to make the progressive calculation fast convergence, the step size parameter μ is added to adjust the convergence rate, and the next set of coefficients $W_{k+1}$ is calculated by the current filter coefficient $W_{k}$ [13].

(8) $$W_{k+1}=W_{k}+\mu \nabla_{k}=W_{k}-2\mu e\left(k\right)X_{k}$$

when using the adaptive filter in practice, attention should be paid to the selection of the coefficient length *L* and the adaptive step size parameter μ. Generally, *L* is not less than the number of non-zero eigenvalues of the autocorrelation matrix of the input signal. The range of μ is $0<\mu <\frac{1}{LS_{\max}}$, where $S_{\max}$ is the maximum value of the power spectral density of the input signal [14]. If the value of μ is too small, the convergence speed will be slowed down. If the value of μ is too large, the error will increase. In order to increase the convergence speed, μ can be set to a variable value, the large value is used to quickly approach the optimal value area, and then the small step size is used to approach the optimal value.

NLMS algorithm: x(n) is proportional to the steady-state error and the step size parameter μ is inversely proportional to the steady-state error. As x(n) increases, a large filter steady-state error is generated. It is necessary to change the step size parameter μ to reduce the value of the steady-state error. Based on the LMS algorithm, the NLMS algorithm “normalizes” the fixed step size parameters by the energy of the input signal x(n), and continuously changes the step size parameter [15]. Therefore, the NLMS algorithm has higher adaptability, flexibility, and stability than the LMS algorithm. The description of the NLMS algorithm [13,16] is as follows:

(9) $$W_{k+1}=W_{k}+\mu \nabla_{k}=W_{k}+\frac{\alpha }{{∥x(n)∥}^{2}+\beta }e\left(k\right)X_{k}$$

where ${∥x(n)∥}^{2}$ is the Euclidean norm square of the tap input signal x(n) and α is the fixed step size parameter, 0<α<2. In order to prevent the input signal x(n) from being too small, then ${∥x(n)∥}^{2}$ is too small, and the step size parameter is too large, so that the algorithm cannot converge, and a positive constant β is introduced, 0<β<1.

## 4. Experiments and Results

According to Figure 1, the corresponding optical system structure is designed, and the valid signal is set to a sine wave, and the noise signal is set to Gaussian white noise. The sampling rate of the system signal is set to 40 kHz and the number of samples is 20,000. The adaptive filter length is set to 128, and there are two kinds of adaptive algorithms. One is the LMS algorithm, the step size parameter μ is set to a variable value and automatically adjusted to the optimal step size. The other is the NLMS algorithm, the fixed step size parameters α are tested in advance, 0.2 is the best value, and the step size parameter changes with the energy of x(n).

### 4.1. Power Spectrum with a Signal Frequency of 1 kHz

**Figure 4:**
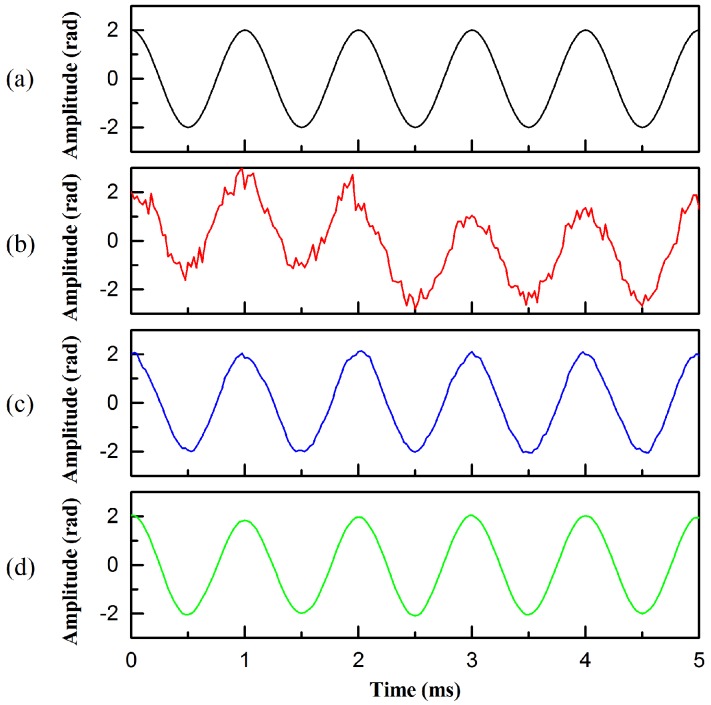
Demodulated signal before and after filtering. (**a**) The original signal, (**b**) the signal before filtering, (**c**) the signal filtered by normalized least mean square (NLMS) algorithms, and (**d**) the signal filtered by least mean square (LMS) algorithms.

In the case where the signal frequency is 1 kHz, the demodulation effect diagram of the sensing signal of the fiber-optic hydrophone after adaptive filtering is shown in Figure 4, and the remaining test results are similar to those in Figure 4. It can be seen from Figure 4 that after the adaptive filtering of the two algorithms, the demodulated signals are obviously improved, and the amplitude is stable in the range of 2 rad. However, in comparison, the LMS algorithm has a better filtering effect and a smoother signal curve.

**Figure 5:**
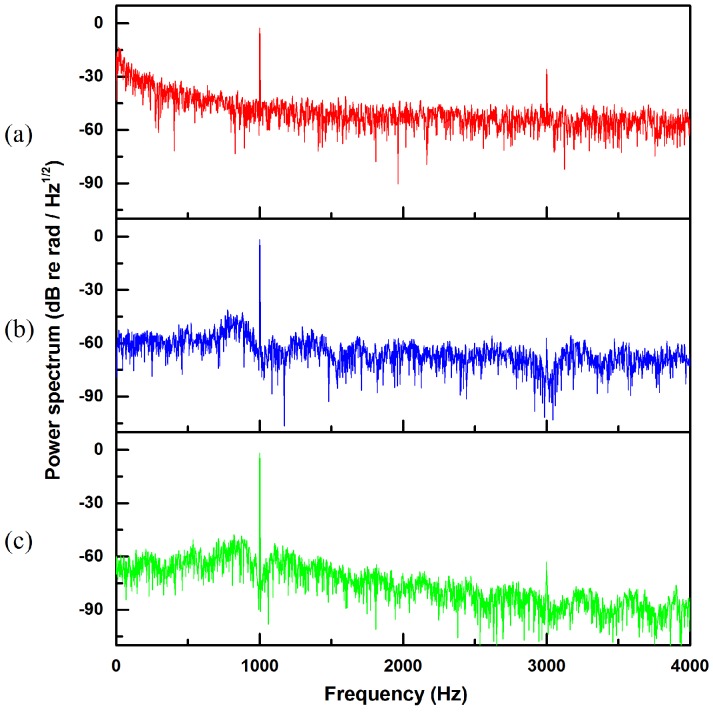
Power spectrum before and after filtering. (**a**) Power spectrum of the signal before filtering, (**b**) power spectrum of the signal filtered by NLMS algorithms, and (**c**) power spectrum of the signal filtered by LMS algorithms.

Figure 5 shows the power spectrum of the demodulated signal in this case. It can be seen from Figure 5 that after two kinds of adaptive filtering, the noise and the harmonic signal are significantly reduced, and the noise near the 1 KHz signal is reduced by about 20 dB. Among them, the reason why the even-multiplied frequency harmonic signals disappears is that in the demodulation process, each signal can be expanded into a Fourier series, $f\left(t\right)=\frac{a_{0}}{2}+\sum_{n=1}^{\infty }\left(a_{n}\cos n\omega_{0}t+b_{n}\sin n\omega_{0}t\right)$, $\omega_{0}=2\pi /T$; n=1,2,3,⋯. After the demodulation algorithm operation, the even-multiplied frequency harmonic signals cancel each other out. The odd-multiplied frequency harmonic signals are obviously reduced by adaptive filtering. From the figure, it can be seen that the triple-frequency harmonic signals are reduced by about 30 dB, and the other odd-multiplied frequency harmonic signals are similar. However, overall, the LMS algorithm’s effect of reducing noise and harmonics is better than that of NLMS.

### 4.2. In the Case of the Different Signal Frequencies

Generally, the evaluation indexes of the signal include total harmonic distortion (THD), signal-to-noise ratio (SNR), and power spectrum. Among them, the size of THD reflects the degree of the signal’s harmonic distortion. The smaller the value, the less the harmonic signal component, and the smaller the distortion. THD is usually expressed as a percentage. SNR is the ratio of useful signal power to noise signal power. In order to facilitate the calculation and highlight the difference between the results before and after the filtering, the above evaluation indicators are expressed in decibels (dB).

**Figure 6:**
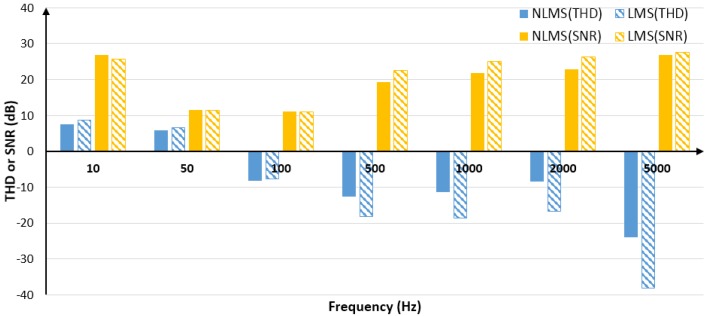
Comparison of filtering effects of two algorithms in terms of total harmonic distortion (THD) and signal-to-noise ratio (SNR).

In order to observe the adaptive filtering performance of the LMS algorithm and the NLMS algorithm the noise intensity is constant and the signal frequency is different. By adjusting the frequency of the valid signal, setting the signal amplitude to 2 V, the test results with the same noise intensity but different signal frequencies are obtained. Since THD and SNR vary with the signal, the average of 100 sets of THD and 100 sets of SNR is calculated to reflect the overall filtering performance in terms of THD and SNR. The difference before and after filtering is taken as the improvement extent, and the comparison of the filtering effects of the two algorithms is shown in Figure 6. See Table A1 and Table A2 of Appendix A for detailed results.

It can be seen from Figure 6 that the overall performance of the adaptive filtering of the LMS algorithm and NLMS algorithm is good, but the filtering effect of the two algorithms on the harmonics within 50 Hz is poor. As the low-frequency signal has many harmonics, filtering out the harmonics and filtering out some of the effective signals results in an increase in THD. However, when the signal frequency is above 100 Hz (excluding 100 Hz), the effect of harmonic filtering is obvious, mainly because the harmonic signal of the high frequency signal exceeding the sampling frequency cannot be collected. Therefore, the THD drop of the high-frequency signal is more prominent, such as 5000 Hz, and it can be seen that the degree of reduction of the LMS algorithm is more obvious, which is about 14 dB higher than the NLMS algorithm.

After the signal is adaptively filtered, both algorithms significantly improve the SNR. Above 100 Hz, the higher the signal frequency, the better the SNR improvement effect. However, the improvement effect of the LMS algorithm is about 3 dB higher than the NLMS algorithm.

### 4.3. In the Case of Different Signal Amplitudes

**Figure 7:**
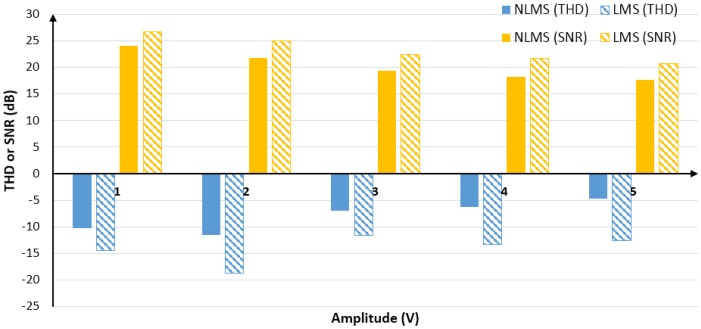
Comparison of filtering effects of two algorithms in terms of THD and SNR.

To observe the adaptive filtering performance of the LMS algorithm and the NLMS algorithm when the noise intensity is constant and the signal amplitudes is different, by adjusting the intensity of the valid signal, setting the signal frequency to 1KHz, the test results with the same noise intensity but different signal amplitudes. The comparison of the filtering effects of the two algorithms is shown in Figure 7. See Table A3 and Table A4 of Appendix A for detailed results.

As seen in Figure 7, both algorithms have achieved good filtering results under different signal amplitudes. Among them, the filtering effect of the LMS algorithm was significantly better than that of the NLMS algorithm. In terms of THD, the former was about 6 dB better than the latter on the average, and the former was about 3 dB better than the latter in terms of SNR.

In addition to the above comparison experiments, comparative experiments were also conducted in the case where the signal frequency is constant and the noise intensity is different. As the number of comparison experiments was limited, it is no longer listed. However, the experimental results also show that the filtering effect of the LMS algorithm is better. Although they have advantages and disadvantages in terms of SNR improvement, the LMS algorithm performed better in THD, and the THD reduction was about 7 dB higher than the NLMS algorithm.

The reason why the two algorithms differed in filtering performance was mainly due to the selection of the step size parameter μ. The LMS algorithm first approached the optimal value region with a large step size, and then approached the optimal value with a small step size. When the signal fluctuated greatly, the value of μ gradually approached the optimal value, so loss of the valid signal was small. However, the NLMS algorithm changed according to the change of energy of the input signal x(n). When the input signal fluctuated greatly, the value of μ changed more sharply, and the resulting signal error became larger, especially in the case of low energy input signal. In addition, as the ratio of the sampling rate to the signal frequency became smaller and smaller, the difference between each adjacent signal point also became larger, which also caused a loss of valid signal and reduction of the filtering effect.

## 5. Conclusions

Adaptive filtering could adjust its own parameters with signal changes to achieve the best filtering effect. It could replace the classical filters with fixed parameters, poor adaptability and robustness, and can be introduced into the signal processing of sensor units, arrays, and even networks. In this paper, by adding a reference interferometer with the same parameters as the signal interferometer as the reference channel, the effects of the adaptive filtering scheme based on LMS algorithm and NLMS algorithm were tested respectively. The results showed that the LMS algorithm and NLMS algorithm not only reduced harmonic distortion and noise, but also improved the total harmonic distortion and signal-to-noise ratio. However, compared with the NLMS algorithm, the LMS algorithm had better improvement of total harmonic distortion and signal-to-noise ratio, and a better filtering effect and smaller calculation amount. Therefore, from the perspective of real-time processing, computational complexity, and filtering effect, the adaptive filtering scheme based on LMS algorithm was more suitable for the noise reduction processing of interferometric fiber-optic hydrophone sensing signals.

## Abbreviations

The following abbreviations are used in this manuscript:

LMS	least mean square
NLMS	normalized least mean square
PGC	phase generation carrier
PZT	piezoelectric ceramic transducer
PD	photoelectric detector
AC	alternating current
THD	total harmonic distortion
SNR	signal-to-noise ratio

## Appendix A

The test results of the NLMS and LMS algorithms under the same noise intensity and signal amplitude but different signal frequencies are shown in Table A1 and Table A2.

**Table A1:** Test results of NLMS algorithm with different signal frequencies (unit: dB).

Frequency	THD (Before)	THD (After)	Difference	SNR (Before)	SNR (After)	Difference
10 Hz	−14.26	−6.72	7.54	−16.66	10.2	26.86
50 Hz	−14.48	−8.62	5.86	−5.03	6.53	11.56
100 Hz	−1.5	−9.72	−8.22	−2.15	8.85	11
500 Hz	−8.35	−21.05	−12.7	−0.6	18.76	19.36
1000 Hz	−11.23	−22.69	−11.46	−0.97	20.81	21.78
2000 Hz	−14.07	−22.46	−8.39	−2.22	20.72	22.94
5000 Hz	−1.34	−25.39	−24.05	−7.15	19.67	26.82

**Table A2:** Test results of LMS algorithm with different signal frequencies (unit: dB).

Frequency	THD (Before)	THD (After)	Difference	SNR (Before)	SNR (After)	Difference
10 Hz	−14.26	−5.68	8.58	−16.66	8.96	25.62
50 Hz	−14.48	−8.01	6.47	−5.03	6.37	11.4
100 Hz	−1.5	−9.23	−7.73	−2.15	8.8	10.95
500 Hz	−8.35	−26.52	−18.17	−0.6	22	22.6
1000 Hz	−11.23	−29.9	−18.67	−0.97	24.05	25.02
2000 Hz	−14.07	−30.72	−16.65	−2.22	24	26.22
5000 Hz	−1.34	−39.39	−38.05	−7.15	20.51	27.66

The test results of the NLMS and LMS algorithms under the same noise intensity and signal frequency but different signal amplitudes are shown in Table A3 and Table A4.

**Table A3:** Test results of NLMS algorithm with different signal amplitudes (unit: dB).

Amplitude	THD (Before)	THD (After)	Difference	SNR (Before)	SNR (After)	Difference
1 V	−12.14	−22.32	−10.18	−4.71	19.38	24.09
2 V	−11.23	−22.69	−11.46	−0.97	20.81	21.78
3 V	−15.08	−22.07	−6.99	4.51	23.87	19.36
4 V	−14.71	−21	−6.29	6.21	24.49	18.28
5 V	−17.85	−22.49	−4.64	7.59	25.2	17.61

**Table A4:** Test results of LMS algorithm with different signal amplitudes (unit: dB).

Amplitude	THD (Before)	THD (After)	Difference	SNR (Before)	SNR (After)	Difference
1 V	−12.14	−26.54	−14.4	−4.71	22.01	26.72
2 V	−11.23	−29.9	−18.67	−0.97	24.05	25.02
3 V	−15.08	−26.68	−11.6	4.51	26.99	22.48
4 V	−14.71	−28.02	−13.31	6.21	27.87	21.66
5 V	−17.85	−30.47	−12.62	7.59	28.29	20.7
